# Genetic screening: The vista of genomic medicine

**DOI:** 10.4103/0975-7406.76487

**Published:** 2011

**Authors:** Rajiv Saini, Santosh Saini, Gagan Saini

**Affiliations:** Department of Periodontology and Oral Implantology, Rural Dental College, Loni, Maharashtra, India; 1 Department of Microbiology, Rural Medical College, Loni, Maharashtra, India; 2 Research Fellow, Massachusetts Institute of Technology, Boston, USA

**Keywords:** Genetics, genetic testing, genomic, therapy

## Abstract

The accelerating development of biochemical and DNA-based diagnostic tests for human genetic conditions in the last decade has engendered a revolution in genetic diagnosis. Both genetic testing and genetic screening involve the same testing processes to examine an individual’s chromosomes, DNA, or the biochemical product of a gene, typically a protein to confirm or refute a suspected chromosomal, DNA, or gene product change. The identification of genetic disorders, and the potential for developing a therapy, is a powerful force in genetics and medicine.

Genetic testing is the use of laboratory tests to determine the genetic status of individuals already suspected to be at high risk for a particular genetic disorder based on family history or a positive screening test, and genetic testing and screening are similar in that both involve the use of laboratory tests to reveal the presence of disease-causing genes. The accelerating development of biochemical and DNA-based diagnostic tests for human genetic conditions in the last decade has engendered a revolution in genetic diagnosis. Finally, the pace of development and application of DNA and biochemical genetic tests and their acceptance by the public may be accelerated by the recent widespread media coverage of the work of human geneticists.[1] A genetic test is the analysis of human DNA, RNA, chromosomes, proteins, and certain metabolites in order to detect heritable disease-related genotypes, mutations, phenotypes, or karyotypes for clinical purposes.[2] Genetic tests also have varied rationale, including the analysis of genetic disease in newborns, children, and adults; the recognition of potential health risks; the prediction of drug responses; and the evaluation of risks to future children. Bioinformatics is a rapidly emerging field of biomedical research. A flood of large-scale genomic and postgenomic data means that many of the challenges in biomedical research are now the challenges in computational science. Clinical informatics has long developed methodologies to improve biomedical research and clinical care by integrating experimental and clinical information systems. The informatics revolution in both bioinformatics and clinical informatics will eventually change the current practice of medicine, including diagnostics, therapeutics, and prognostics. Postgenome informatics, powered by high-throughput technologies and genomic-scale databases, is likely to transform our biomedical understanding forever, in much the same way that biochemistry did a generation ago.[3]

## Characteristics of Genetic Testing

In gene tests, scientists scan a patient’s DNA sample for mutated sequences. A DNA sample can be obtained from any tissue, including blood. For some types of gene tests, researchers design short pieces of DNA called probes, whose sequences are complementary to the mutated sequences. These probes will seek their complement among the 3 billion base pairs of an individual’s genome. If the mutated sequence is present in the patient’s genome, the probe will bind to it and flag the mutation. Another type of DNA testing involves comparing the sequence of DNA bases in a patient’s gene to a normal version of the gene. Cost of testing depends on the sizes of the genes and the numbers of mutations tested. Both genetic testing and genetic screening involve the same testing processes to examine an individual’s chromosomes, DNA, or the biochemical product of a gene, typically a protein to confirm or refute a suspected chromosomal, DNA, or gene product change. Genetic screening is done for a particular condition in individuals, groups, or populations without family history of the condition, and genetic testing is done for a particular condition where an individual is suspected of being at increased risk due to their family history or the result of a genetic screening test.

## Advantages and Scope

Advances in genome technology and other fruits of the Human Genome Project are playing a growing role in the delivery of health care. With the development of new technologies and opportunities for large-scale analysis of the genome, transcriptome, proteome, and metabolome, the genome sciences are poised to have a profound impact on clinical medicine. Cancer prognostics will be among the first major test cases for a genomic medicine paradigm, given that all cancer is caused by genomic instability, and microarrays allow assessment of patients’ entire expressed genomes.[4] Genomic medicine aims to revolutionize health care by applying our growing understanding of the molecular basis of disease. Research in this arena is data intensive, which means data sets are large and highly heterogeneous. To create knowledge from data, researchers must integrate these large and diverse data sets. This presents daunting informatic challenges such as representation of data that is suitable for computational inference (knowledge representation), and linking heterogeneous data sets (data integration). Fortunately, many of these challenges can be classified as data integration problems, and technologies exist in the area of data integration that may be applied to these challenges.[5]

## Genetic Diagnosis

The diagnostic techniques outlined briefly above are a powerful new tool in all genetics, but most especially in the arena of human genetics. By application of these tools of biotechnology, and other techniques, molecular biologists and geneticists are providing a basis on which to make a genetic diagnosis. The identification of genetic disorders, and the potential for developing a therapy, is a powerful force in genetics and medicine. Diagnostic techniques can be used to identify specific proteins or fragments of DNA. These techniques can be used to detect viruses of animal and plant diseases, to detect harmful substances in the environment, or to match the DNA left at a crime scene to a possible criminal. Diagnostic techniques often utilize DNA probes, RFLP (restriction fragment length polymorphism) analysis, and mono- or polyclonal antibodies. Advances in genomics have led to mounting expectations in regard to their impact on health care and disease prevention. In light of this fact, a comprehensive research agenda is needed to move human genome discoveries into health practice in a way that maximizes health benefits and minimizes harm to individuals and populations. We present a framework for the continuum of multidisciplinary translation research that builds on previous characterization efforts in genomics and other areas in health care and prevention. The continuum includes four phases of translation research that revolve around the development of evidence-based guidelines. Phase 1 translation (T1) research seeks to move a basic genome-based discovery into a candidate health application (e.g., genetic test/intervention). Phase 2 translation (T2) research assesses the value of a genomic application for health practice leading to the development of evidence-based guidelines. Phase 3 translation (T3) research attempts to move evidence-based guidelines into health practice, through delivery, dissemination, and diffusion research. Phase 4 translation (T4) research seeks to evaluate the “real world” health outcomes of a genomic application in practice.[6]

## Types of Genetic Testing

Genetic testing is a complex process, and the results depend both on reliable laboratory procedures and accurate interpretation of results. Tests also vary in sensitivity, that is, their ability to detect mutations or to detect all patients who have or will get the disease. Interpretation of test results is often complex even for trained physicians and other health care specialists. When interpreting the results of any genetic test, one must take into account the probability of false positive or false negative test results. Special training is required to be able to analyze and convey information about genetic testing to affected individuals and their families. Available types of genetic testing are listed in Table 1

**Table 1:** Types of genetic test

Genetic test	Feature
Newborn screening	Newborn screening is used just after birth to identify genetic disorders that can be treated early in life
	The routine testing of infants for certain disorders is the most widespread use of genetic testing
Diagnostic testing	Diagnostic testing is used to diagnose or rule out a specific genetic or chromosomal condition
	In many cases, genetic testing is used to confirm a diagnosis when a particular condition is suspected based on physical mutations and symptoms
	Diagnostic testing can be performed at any time during a person’s life, but is not available for all genes or all genetic conditions
Carrier testing	Carrier testing is used to identify people who carry one copy of a gene mutation that, when present in two copies, causes a genetic disorder
	This type of testing is offered to individuals who have a family history of a genetic disorder and to people in ethnic groups with an increased risk of specific genetic conditions
	If both parents are tested, the test can provide information about a couple’s risk of having a child with a genetic condition
Prenatal testing	Prenatal testing is used to detect changes in a fetus’s genes or chromosomes before birth
	This type of testing is offered to couples with an increased risk of having a baby with a genetic or chromosomal disorder In some cases, prenatal testing can lessen a couple’s uncertainty or help them decide whether to abort the pregnancy
Preimplantation genetic diagnosis	Genetic testing procedures that are performed on human embryos prior to the implantation as part of an *in vitro* fertilization procedure
Predictive and presymptomatic testing	Predictive and presymptomatic types of testing are used to detect gene mutations associated with disorders that appear after birth, often later in life
	These tests can be helpful to people who have a family member with a genetic disorder, but who have no features of the disorder themselves at the time of testing. Predictive testing can identify mutations that increase a person’s chances of developing disorders with a genetic basis, such as certain types of cancer
Forensic testing	Forensic testing uses DNA sequences to identify an individual for legal purposes
	This type of testing can identify crime or catastrophe victims, rule out or implicate a crime suspect, or establish biological relationships between people (e.g., paternity)
Parental testing	This type of genetic test uses special DNA markers to identify the same or similar inheritance patterns between related individuals
Research testing	Research testing includes finding unknown genes, learning how genes work, and advancing our understanding of genetic conditions The results of testing done as part of a research study are usually not available to patients or their healthcare providers
Pharmacogenomics	This type of genetic testing determines the influence of genetic variation on drug response

## Issues and Ethics

A comprehensive study of the significance and varieties of genetic discrimination is critical to design strategies to ensure the ethical and appropriate use of genetic testing in the future. The dominant theme noted in the responses in this study is that genetic conditions are regarded by many social institutions as extremely serious, disabling, or even lethal conditions without regard to the fact that many individuals with “abnormal” genotypes will either be perfectly healthy, have medical conditions which can be controlled by treatment, or experience only mild forms of a disease. As a result of this misconception, decisions by such institutions as insurance companies and employers are made solely on the basis of an associated diagnostic label rather than on the actual health status of the individual or family. The appropriate use of genetic testing information to restrict or limit access to public entitlements such as health care or employment has not been established and may not exist. The cost of such labeling is magnified by the fact that errors in testing and interpretation do occur.[1] The three basic components in genetic screening, that is, ethical, legal, and social issues, are to be considered and these genetic tests have to be performed with privacy, informed consent, and confidentiality. This brief discussion illustrates public expectations and fears about the effect of genomics, challenges to the goals of antidiscrimination laws and to the nature of the physician–patient relationship, and the contrasting perspectives and legal rules that apply to personal medical care and public health. Acknowledgment and examination of these complex issues are critical for identifying the appropriate ethical principles that should be applied and for creating the necessary legislative and regulatory responses.[7]

## Conclusion

Given this situation – powerful and attractive new techniques, social and economic forces pressing for their application, and an incomplete understanding of the potential negative social and personal consequences of genetic testing – concern about the burdens engendered by widespread utilization of genetic tests seems justified.[1] Genetic testing offers important opportunities for diagnosis and assessment of genetic risk. The sensitivity of tests for rare conditions will continue to improve as additional causative mutations are identified. Genetic tests are available to determine the risk of common diseases, but these often have limited predictive values. Evaluating the clinical usefulness of these tests will require a careful assessment of the risks and benefits of testing; the availability of specific measures to reduce risk in genetically susceptible people will be a major consideration.[2] With the sequencing of the human genome only months from its finish, the practice of medicine has now entered an era in which the individual patient’s genome will help determine the optimal approach to care, whether it is preventive, diagnostic, or therapeutic. Genomics, which has quickly emerged as the central basic science of biomedical research, is poised to take center stage in clinical medicine as well.[8] As new genetic tests emerge, their translation into practice will depend not only on their performance based on laboratory standards, but also on their ability to enhance prevention or assist clinicians in diagnosing and treating patients.[9] Challenges of translating pharmacogenomics into clinical practice included ethical, social, legal, and economic issues.[10] There are many barriers to implementation of genetic medicine, including the cost of testing, the genetic literacy of patients and health care providers, and concerns about genetic discrimination; however, health care providers and patients must have realistic expectations about its predictive power and current limitations.[11] Thus it can be concluded that genetic testing is a complex process, and the results depend both on reliable laboratory procedures and accurate interpretation of results.
